# Chemotherapy sequential disitamab vedotin in combination with tislelizumab as systemic therapy of upper tract urothelial carcinoma with cutaneous metastasis: A case report and literature review

**DOI:** 10.1097/MD.0000000000046529

**Published:** 2026-01-09

**Authors:** Kangyu Liu, Ming Zheng, Lei Li, Mingyue Liang, Qingyi Zhu, Min Gu, Baixin Shen

**Affiliations:** a Department of Urology, The Second Affiliated Hospital of Nanjing Medical University, Nanjing, China; b Department of Plastic Surgery, The Second Affiliated Hospital of Nanjing Medical University, Nanjing, China.

**Keywords:** cutaneous metastasis, disitamab vedotin, kidney, tislelizumab, upper tract urothelial carcinoma

## Abstract

**Rationale:**

To investigate the clinical features, management strategies, and prognosis of the rare condition of upper tract urothelial carcinoma (UTUC) with skin metastasis, with emphasis on the efficacy and safety of disitamab vedotin combined with tislelizumab in systemic therapy, we performed a literature search and selection. We searched PubMed, Embase, and Web of Science for English-language publications from January 1, 1990, through December 10, 2024 (last search December 10, 2024). Predefined terms combined “upper tract urothelial carcinoma” or “UTUC” with “skin,” “cutaneous,” “abdominal wall,” or “port-site,” and with “metastasis,” “implantation,” or “seeding.” Inclusion criteria were human case reports or small series with histologically confirmed UTUC and cutaneous involvement. We excluded non-UTUC primaries, noncutaneous metastases, pure reviews without primary cases, duplicate reports, and articles lacking extractable case-level clinical or pathologic data. Two reviewers independently screened records and extracted data (patient and tumor features, site and timing of cutaneous involvement, other metastases, postdiagnosis treatment, and outcomes), resolving discrepancies by consensus. Owing to rarity and heterogeneity, we performed a qualitative descriptive synthesis without meta-analysis.

**Patient concerns:**

A patient developed a progressively enlarging cutaneous mass on the abdominal wall at a prior laparoscopic port site following renal cyst surgery.

**Diagnoses:**

Biopsy confirmed metastatic poorly differentiated urothelial carcinoma. On April 27, 2023, the patient underwent robot-assisted radical nephrectomy with abdominal wall tumor resection and latissimus dorsi myocutaneous flap transfer. Postoperative pathology revealed high-grade urothelial carcinoma with human epidermal growth factor receptor 2 (+++) expression.

**Interventions:**

Gemcitabine/cisplatin was administered initially; however, due to intolerance to cisplatin-based chemotherapy, sequential disitamab vedotin (120 mg every 3 weeks) plus tislelizumab (200 mg every 3 weeks) was initiated on November 23, 2023.

**Outcomes:**

At 24-month postoperative follow-up, there was no evidence of local recurrence at the surgical site.

**Lessons:**

Cutaneous metastasis in UTUC is rare and portends a poor prognosis. Radical surgery combined with systemic therapy remains the cornerstone of care. In this case, disitamab vedotin plus tislelizumab, used as a postoperative sequential systemic regimen, demonstrated favorable safety and achieved a 24-month recurrence-free interval after discontinuation of gemcitabine/cisplatin due to intolerance, with manageable adverse effects. This combination may represent a potential treatment option for metastatic UTUC, although its efficacy requires validation in larger cohorts.

## 1. Introduction

Upper tract urothelial carcinoma (UTUC) is a highly malignant tumor, accounting for 5% to 10% of urothelial carcinomas.^[1]^ The primary symptoms include gross hematuria and microscopic hematuria, often accompanied by renal discomfort, such as lower back pain.^[2]^ Common metastatic sites for UTUC include lymph nodes, lungs, liver, and bones.^[3]^ However, cutaneous metastases from UTUC are exceedingly rare, and due to the limited number of reported cases, their precise incidence remains unclear.^[4–8]^ In 2023, the Department of Urology at the Second Affiliated Hospital of Nanjing Medical University treated a case of UTUC with skin metastasis. This report combines clinical data with a review of the relevant literature to elucidate the diagnosis and treatment of UTUC with cutaneous metastases. Additionally, it discusses the role of disitamab vedotin combined with tislelizumab in postoperative adjuvant therapy for UTUC, providing valuable insights into this rare condition.

## 2. Case presentation

**Figure 1.**
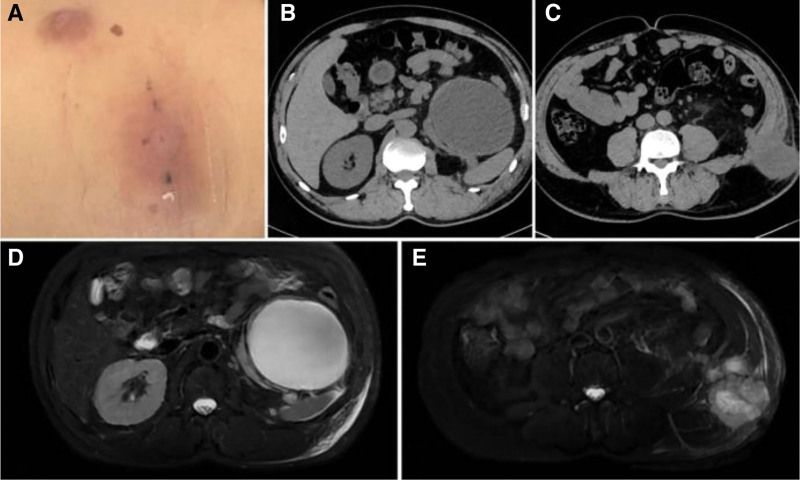
(A) Appearance of skin mass: redness around 2 sites of the original laparoscopic ventral incision on the left abdominal wall, hard texture beneath the incision; (B, C) preoperative contrast-enhanced CT: cystic low-density shadow in the left kidney measuring approximately 10.7 × 9.5 cm, with clear boundaries and localized dense shadows at the margin; multiple masses with heterogeneous density posterior to the left kidney and in the left posterior abdominal wall, the largest measuring approximately 4.3 × 4.8 cm; abdominal wall lesions involving the left latissimus dorsi, internal oblique, and transversus abdominis muscles; (D, E) preoperative MRI appearance: oval-shaped cystic lesion in the left kidney showing non-enhancing long T2/long T1 signal, clear boundaries; multiple masses and nodular lesions with long T2/long T1 signal in the subcutaneous tissue of the left posterior abdominal wall, hyperintense on DWI, irregular in morphology.

A 60-year-old man presented to our hospital on April 17, 2023, with a mass in the left lumbar region. In November 2022, the patient undergone laparoscopic decompressive deroofing of a left renal cyst at a local hospital. Soon after discharge, the patient noted a subincisional mass that gradually enlarged. A CT scan at the local hospital revealed a large left renal cystic lesion (108 × 85 × 140 mm) with multiple masses in the left perirenal space and posterior abdominal wall. Physical examination showed erythema surrounding 2 prior port-site scars on the left abdominal wall, mild warmth, and a firm, poorly mobile subincisional mass with indistinct margins and no marked tenderness. (Fig. 1A).

**Figure 2.**
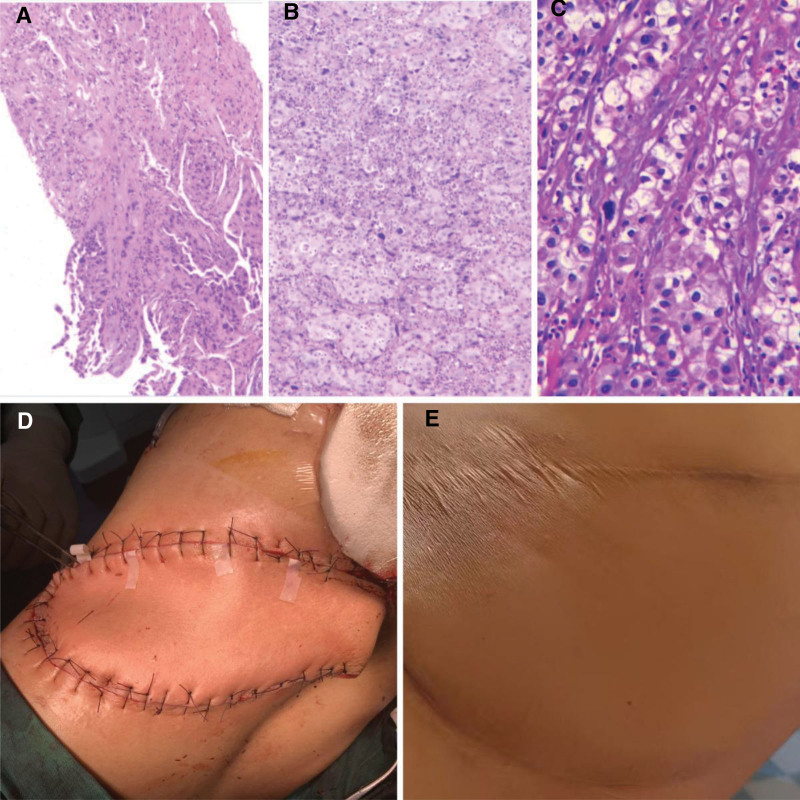
Skin mass and renal pathology, and left latissimus dorsi myocutaneous flap transfer. Needle biopsy pathology of the skin mass (A) and excisional biopsy pathology (B) show metastatic poorly differentiated carcinoma in the stroma, favoring a urological (kidney, ureter, bladder, etc) origin (HE 100×); (C) renal tumor pathology showing tumor cells arranged in nests, with pleomorphic nuclei and eosinophilic cytoplasm. Chronic inflammatory cell infiltration within fibrous septa (HE 400×); (D) left latissimus dorsi myocutaneous flap transfer: resection around the tumor to subcutaneous tissue within a safe margin; elliptical incision approximately 20 cm long made in the lumbar region; complete resection of the abdominal wall mass; latissimus dorsi myocutaneous flap approximately 30 × 15 cm harvested at the level of the 12th rib; flap transferred to the abdominal wall defect and sutured in place; back defect covered with a skin graft from surrounding skin; (E) postoperative appearance of the lumbar skin.

**Figure 3.**
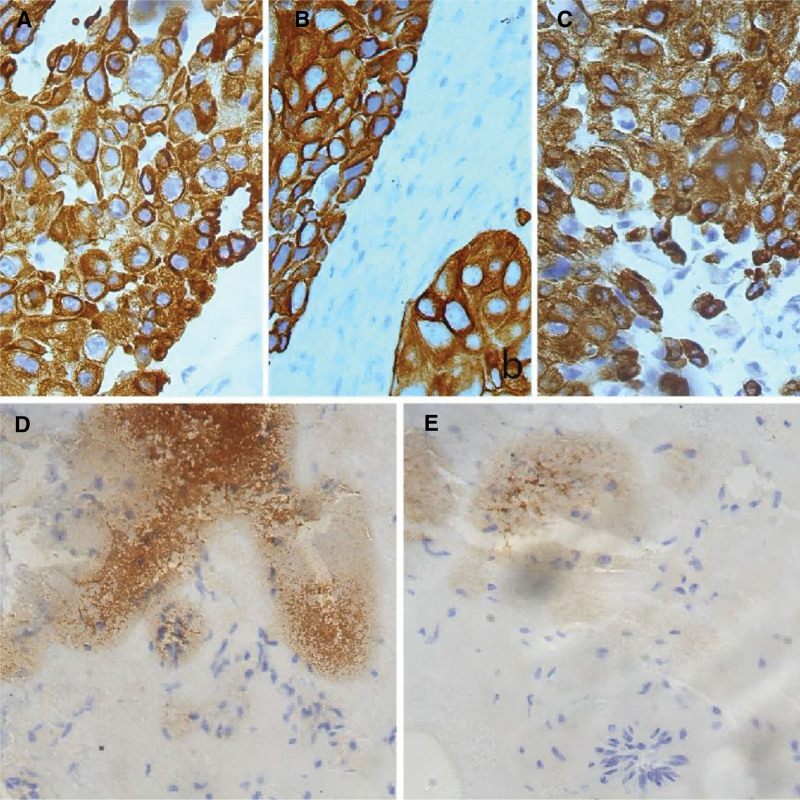
Immunohistochemistry analysis. (A) Cutaneous metastasis: CK7 positivity (400×); (B) abdominal wall mass: CK7 positivity (400×); (C) renal tumor: CK7 positivity (400×); (D) renal tumor: HER2 positivity (400×); (E) renal tumor: PD-1 positivity (400×).

**Figure 4.**
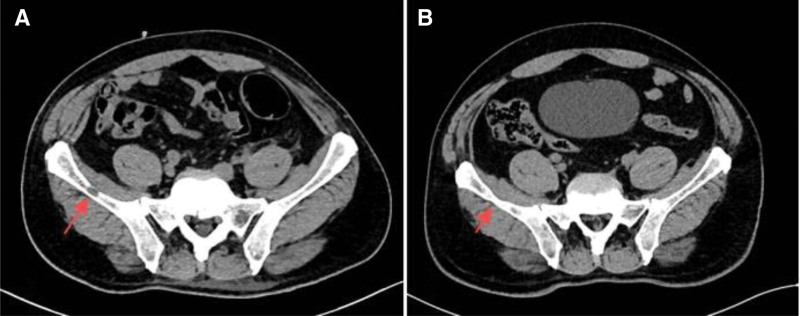
CT appearance of iliac bone metastasis before and after treatment. (A) Follow-up CT after 6 cycles of gemcitabine–cisplatin chemotherapy showing a newly developed osteolytic metastasis in the right iliac bone; (B) follow-up CT in April 2025 postoperatively showing post-treatment changes in the iliac bone metastasis.

On the same day at our hospital (April 17, 2023), abdominal contrast-enhanced CT demonstrated a cystic low-attenuation lesion in the left kidney with clear boundaries. Multiple masses with heterogeneous density were seen posterior to the left kidney and in the left posterior abdominal wall, involving the left latissimus dorsi, internal oblique, and transversus abdominis muscles (Fig. 1B and C). MRI performed the same day (April 17, 2023) revealed multiple metastases in the left perirenal space, retrorenal space, and subcutaneous tissue of the left posterior abdominal wall, suggestive of malignant lesions (Fig. 1D and E). On April 18 and April 21, 2023, CT-guided biopsies were obtained from the indurated erythematous cutaneous lesion at the prior laparoscopic port site on the left abdominal wall and from the deeper abdominal wall mass, respectively, both revealing poorly differentiated carcinoma favoring a urological (kidney/ureter/bladder) origin (Fig. 2A and B). Immunohistochemistry (IHC) on the cutaneous biopsy showed GATA3 (partial+), p63 (partial+), and CK7 (+) (Fig. 3A); melanocytic markers (S-100/Melan-A) were negative, and RCC markers (RCC antigen/CAIX) were negative. The abdominal wall core biopsy showed CK7 (+), GATA3 (partial+), p63 (−), and CK5/6 (+), with RCC markers negative (Fig. 3B). After multidisciplinary discussion, the patient underwent robot-assisted left radical nephrectomy with en bloc abdominal wall tumor resection and latissimus dorsi myocutaneous flap transfer on April 27, 2023 (Fig. 2D). Grossly, a cystic mass at the upper pole measured approximately 9 × 8.5 × 5 cm, partially abutting the renal pelvis. The cystic surface was rough with several mural projections (largest 1.5 × 1 × 0.8 cm). Multiple firm gray-white nodules (0.5–2.0 cm) were present in the adjacent renal parenchyma. Postoperative pathology confirmed high-grade urothelial carcinoma (Fig. 2C). IHC of the nephrectomy tumor showed CK7 (+), p63 (rare+), and CK20 (focal+) (Fig. 3C); GATA3 (−); RCC antigen (−) with focal/partial CAIX and CD10; PAX8 (+) noted. HER2 showed overexpression (IHC 3+) (Fig. 3D). PD-1 was strongly positive (Fig. 3E). Postoperatively, the patient received regular chemotherapy with gemcitabine + cisplatin (cisplatin 120 mg every 4 weeks + gemcitabine 2 g every 4 weeks). During cycle 6, the patient developed severe adverse events, including hematologic toxicity: nadir platelets 44 × 10^9^/L (CTCAE v5.0 grade 3) and hemoglobin 6.7 g/dL (grade 3) on October 09, 2023 and intense vomiting. After packed red blood cell and platelet transfusions, symptoms improved; however, given confirmed multi-lineage myelosuppression, further platinum-based chemotherapy was discontinued. Subsequently, a CT scan in November 2023 showed no obvious recurrence in the left surgical area but revealed a new metastasis in the right iliac bone (Fig. 4A). Therefore, sequential disitamab vedotin plus tislelizumab was initiated on November 23, 2023 (tislelizumab 200 mg every 3 weeks; disitamab vedotin 120 mg every 3 weeks). Following initiation, the patient’s vomiting resolved, and blood cell counts returned to normal levels. The patient received a total of 12 cycles of this therapy. During the 24-month postoperative follow-up period, a follow-up abdominal CT scan in April 2025 showed no recurrence at the surgical site. The right iliac bone lesion showed localized thinning and distortion, consistent with post-treatment changes (Fig. 4B). For readability, a consolidated timeline of key clinical events, diagnostic procedures, treatments, and follow-up is provided in Table 1.

**Table 1:** Timeline of key clinical events.

Date	Event	Key details/findings
November 2022	Laparoscopic deroofing of left renal cyst (local hospital)	Soon after discharge, a subincisional mass appeared and gradually enlarged. Local CT: large left renal cystic lesion (108 × 85 × 140 mm) with multiple masses in the left perirenal space and posterior abdominal wall.
Apri 17, 2023	Presentation to our hospital; imaging work-up	Physical exam: induration and erythema around 2 prior port-site scars. Contrast-enhanced CT: left renal cystic low-attenuation lesion; multiple masses posterior to the left kidney and left posterior abdominal wall, involving latissimus dorsi, internal oblique, and transversus abdominis. MRI: multiple lesions in left perirenal/retrorenal spaces and subcutaneous posterior abdominal wall.
April 18, 2023	CT-guided biopsy: prior port-site cutaneous/subcutaneous lesion	Poorly differentiated carcinoma favoring a urological origin. IHC: CK7 (+), GATA3 (partial+), p63 (partial+); S-100/Melan-A (−); RCC antigen/CAIX (−).
April 21, 2023	CT-guided biopsy: deeper abdominal wall mass	Poorly differentiated carcinoma. IHC: CK7 (+), CK5/6 (+), GATA3 (partial+), p63 (−); RCC markers negative.
April 27, 2023	Robot-assisted left radical nephrectomy with en bloc abdominal wall tumor resection and latissimus dorsi myocutaneous flap transfer	Pathology: high-grade urothelial carcinoma. IHC: 34βE12 (+), CK7 (+), p63 (rare+), CK20 (focal+), GATA3 (−), RCC antigen (−) with focal/partial CAIX and CD10, PAX8 (+). HER2 overexpression (IHC 3+).
Post-op 2023	Adjuvant chemotherapy (GC)	Gemcitabine + cisplatin (cisplatin 120 mg q4w; gemcitabine 2 g q4w).
November 2023 (after 6 cycles)	Restaging CT and tolerance	Severe adverse effects (cytopenia, intense vomiting) → unable to tolerate further cisplatin-based chemotherapy. CT: no local recurrence at the surgical site; new right iliac bone metastasis.
November 23, 2023	Sequential systemic therapy initiated	Disitamab vedotin 120 mg q3w + tislelizumab 200 mg q3w. Vomiting resolved; blood counts normalized.
2023–2024	Course of DV + tislelizumab	Total of 12 cycles administered.
April 2025 (24 mo post-op)	Latest follow-up status (as stated in manuscript)	No evidence of local recurrence at the surgical site during 24-mo postoperative follow-up.

## 3. Discussion

Cutaneous metastasis is exceedingly rare in UTUC and typically represents a manifestation of advanced-stage disease.^[6]^ We conducted a structured review of English-language literature from 1990 to 2024 (PubMed, Embase, and Web of Science; last search December 10, 2024), including histologically confirmed UTUC case reports with cutaneous involvement. A total of 9 reports were identified,^[4,6,7,9–14]^ and their main features are summarized in Table 2. In the present case, both the skin mass pathology and postoperative renal mass pathology were consistent with urothelial carcinoma, confirming the diagnosis of UTUC with skin metastasis. Among the 9 reported cases, 4 had survival times of only several months. However, in this case, due to early detection and aggressive treatment, the patient exhibited no other systemic complications during follow-up, and the iliac bone metastasis gradually resolved.

**Table 2:** Summary of case information for upper tract urothelial carcinoma with skin metastasis.

Patient no.	Study	Skin metastasis site	Other metastatic sites	Sex	Age	Treatment for primary tumor	Treatment after diagnosis of skin metastasis	Survival time after diagnosis of skin metastasis
1	Endo R et al^[9]^	Neck, chest	None	F	74	None	Unknown	1 mo
2	Singh P et al^[10]^	Left abdominal wall	Lungs	F	70	RNU	Chemotherapy	1 mo
3	Ghalleb M et al^[11]^	Back, neck	Lungs	M	61	RNU	None	1 mo
4	Truong H et al^[12]^	Left suprapubic region	Unknown	M	59	RNU	Unknown	Unknown
5	Lin CY et al^[6]^	Both arms and upper abdomen	Unknown	F	68	RNU	Unknown	1 mo
6	Mirchia K et al^[13]^	Left posterior abdominal wall	Unknown	F	68	None	Chemotherapy	Several months
7	Pomara G et al^[14]^	Penile coronal sulcus	Lungs	M	76	RNU	Gemcitabine + radiotherapy	Still alive at 8 mo
8	Zirwas MJ et al^[4]^	Right shoulder	Lungs	M	43	RNU	Chemotherapy	Unknown
9	Ando K et al^[7]^	Left chest wall, left axilla, left arm	None	M	67	RNU	Gemcitabine + radiotherapy	Still alive at 10 mo

RNU = radical nephroureterectomy.

Because cutaneous metastasis in UTUC is uncommon, its diagnosis is often overlooked, resulting in delayed treatment and adversely affecting prognosis. Histopathologic confirmation is essential, and subsequent management should be guided by the pathologic diagnosis. Four mechanisms of tumor spread to the skin are recognized: hematogenous spread, lymphatic spread, direct invasion, and iatrogenic implantation during surgery.^[15]^ In specific circumstances, tumor cells can spread to local skin through iatrogenic implantation during surgery (e.g., laparoscopic procedures).^[13,16–18]^ In our patient, the skin metastasis presented as induration around the original laparoscopic incision site on the left abdominal wall, without hematuria, back pain, or systemic symptoms. Given the history of laparoscopic surgery for a “left renal cyst” 3 months prior and imaging findings showing the left renal tumor invading the latissimus dorsi and abdominal wall muscles, it was hypothesized that the metastasis resulted from tumor cell seeding along the original laparoscopic incision tract, rather than being a sign of widespread systemic disease typically reported in the literature.

For high-risk UTUC, radical nephroureterectomy (RNU) the standard of care, encompassing the affected kidney, entire ureter, and a cuff of bladder around the ureteral orifice. The role of surgery in metastatic UTUC has not been fully defined. However, available evidence suggests that selected patients may still benefit from RNU or metastasectomy.^[19]^ Some literature suggests that surgical resection demonstrates better survival outcomes in metastatic UTUC.^[20–22]^ In 1 clinical study predicting overall mortality in 1174 metastatic UTUC patients, RNU treatment was associated with reduced overall mortality.^[23]^ In the cases we reviewed, 2 patients received no treatment for their primary UTUC, while the others developed skin metastases after undergoing RNU.

According to the European Association of Urology guidance and the Chinese Guidelines for Diagnosis and Treatment of Urological and Andrological Diseases,^[1,24]^ platinum-based combination chemotherapy (particularly cisplatin-based) is the 1st-line treatment choice for metastatic UTUC, such as the GC regimen (gemcitabine, cisplatin) or MVAC regimen (methotrexate, vinblastine, doxorubicin, cisplatin). Among the 9 cases we reviewed, 2 patients received no chemotherapy or radiotherapy after diagnosis of skin metastasis and died within 1 to 3 months; 1 received gemcitabine plus radiotherapy and survived beyond 10 months; and 1 received combined chemoradiotherapy and survived beyond 8 months.

After multidisciplinary evaluation, the patient underwent robot-assisted left radical nephrectomy with en bloc abdominal wall tumor resection and latissimus dorsi myocutaneous flap reconstruction. Postoperatively, cisplatin-based chemotherapy with gemcitabine–cisplatin was administered. After 6 cycles of treatment, surveillance CT scan revealed a new metastasis in the right iliac bone. Given the disease status, continuation of chemotherapy was indicated. However, the patient developed severe adverse reactions including cytopenia and vomiting, precluding further cisplatin-based chemotherapy.

Currently, there is a lack of effective 2nd-line treatment options for metastatic UTUC that has failed 1st-line chemotherapy. In recent years, increasing research has shown that immune checkpoint inhibitors (ICIs) have certain efficacy in the 2nd-line treatment of advanced UTUC.^[25]^ Moreover, accumulating evidence from studies in metastatic urothelial carcinoma has demonstrated meaningful therapeutic activity and acceptable safety profiles of ICIs.^[26–29]^ Compared with traditional chemotherapy, ICIs are associated with fewer adverse effects and longer durations of response and survival, making them an important therapeutic option in the post-surgical and advanced settings. These advances highlight the expanding role of immunotherapy in urothelial carcinoma, including UTUC. The growing understanding of immune-related mechanisms has also laid the groundwork for developing new therapeutic combinations in this disease context. Currently, the domestically developed tislelizumab, a PD-1 inhibitor, has been approved by the National Medical Products Administration for 2nd-line treatment of advanced urothelial carcinoma in China.With advances in drug development, the combination of antibody–drug conjugates (ADCs) with ICIs in metastatic urothelial carcinoma patients is garnering increasing attention.^[30–32]^ The mechanism of ADCs involves targeting specific receptors on the tumor cell surface, penetrating the cell membrane, and releasing cytotoxic agents to kill cancer cells, offering higher targeting efficiency and cytotoxic potency. In the EV-103 phase Ib/II multi-cohort study, 45 previously untreated cisplatin-ineligible metastatic urothelial carcinoma patients received enfortumab vedotin (an ADCs) combined with pembrolizumab (a PD-1 inhibitor). Results showed an objective response rate of 73.3%, with a complete response rate of 15.6%, and no significant primary disease progression was observed in any patient.^[33]^ Furthermore, in the NCT03523572 trial,^[34]^ 30 patients with HER2-high metastatic urothelial carcinoma received trastuzumab deruxtecan (an ADCs) combined with nivolumab (a PD-1 inhibitor). The study reported an objective response rate of 36.7%, a complete response of 13.3%, and median duration of response and median overall survival of 13.1 and 11.0 months, respectively. Disitamab vedotin is also an ADC that specifically targets the HER2 receptor on tumor cells. It is indicated for patients with advanced or metastatic urothelial carcinoma who have received prior platinum-containing chemotherapy and have HER2 overexpression. The 2022 CSCO guidelines have included it as a first-line treatment recommendation for metastatic UTUC. In the recent RC48-C017 trial,^[35]^ 44 patients with HER2-positive muscle-invasive bladder cancer received 6 cycles of neoadjuvant disitamab vedotin combined with toripalimab (a PD-1 antibody) every 2 weeks, followed by radical cystectomy. Adjuvant therapy continued postoperatively until disease progression or intolerance. Results showed that the pathological complete response rate in the disitamab vedotin plus PD-1 inhibitor group was 61.3%, and the pathological partial response rate reached 74.2%, demonstrating superior efficacy compared to traditional adjuvant chemotherapy.

After reviewing current international and domestic evidence, relevant guidelines, and our institutional experience, we selected disitamab vedotin plus tislelizumab for this patient. With the patient’s trust and consent, treatment with disitamab vedotin plus tislelizumab was initiated on November 23, 2023. The rationale endorsed by the multidisciplinary team was: HER2 overexpression (IHC 3+) provided a clear biological target for HER2-directed ADC therapy; clinically significant intolerance to cisplatin-based chemotherapy, including grade 3 hematologic toxicity and refractory nausea/vomiting, precluded further platinum exposure; ICIs demonstrate clinically meaningful activity with acceptable safety in metastatic urothelial carcinoma,^[26–29]^ and emerging data support the feasibility of ADC–ICI combinations in urothelial carcinoma;^[33–35]^ and both agents were available at our institution and aligned with the patient’s preference for a regimen with lower emetogenicity. The treatment course has now concluded. During the 24-month postoperative follow-up, no recurrence was observed. A follow-up CT scan showed reduction in the size of the right iliac bone metastasis. This demonstrates the feasibility of ADC combined with ICI therapy for metastatic UTUC.

The prognosis of metastatic UTUC is poor, with a 3-year overall survival rate below 10%.^[21,23]^ Furthermore, due to the scarcity of UTUC cases with skin metastasis, definitive survival rates are currently unavailable. Among the 9 case reports we reviewed, 5 patients survived only 1 to 3 months after detection of cutaneous disease, while the longest reported survival exceeded 10 months. In contrast, the present patient’s survival has exceeded 24 months to date.

## 4. Conclusion

Cutaneous metastasis of UTUC is extremely rare, typically high grade, and associated with unfavorable outcomes. For patients with metastatic UTUC, surgical resection of lesions combined with systemic therapy is a treatment option worth considering. This approach helps control the disease and prolong survival. The combination of disitamab vedotin and tislelizumab has shown efficacy in postoperative adjuvant therapy for patients with UTUC and exhibits relatively high safety. This therapeutic regimen holds promise as an effective treatment for metastatic UTUC. However, large-scale, multicenter randomized controlled trials evaluating ADCs in the systemic therapy for UTUC have not yet been conducted in China. Further prospective studies are warranted to refine treatment strategies and inform clinical decision-making.

## Author contributions

**Conceptualization:** Baixin Shen.

**Methodology:** Lei Li, Qingyi Zhu, Min Gu.

**Supervision:** Mingyue Liang.

**Writing – original draft:** Kangyu Liu.

**Writing – review & editing:** Kangyu Liu, Ming Zheng, Baixin Shen.
